# Critical Role of Exogenous Nitric Oxide in ROCK Activity in Vascular Smooth Muscle Cells

**DOI:** 10.1371/journal.pone.0109017

**Published:** 2014-10-03

**Authors:** Tatsuya Maruhashi, Kensuke Noma, Yumiko Iwamoto, Akimichi Iwamoto, Nozomu Oda, Masato Kajikawa, Takeshi Matsumoto, Takayuki Hidaka, Yasuki Kihara, Kazuaki Chayama, Ayumu Nakashima, Chikara Goto, James K. Liao, Yukihito Higashi

**Affiliations:** 1 Department of Cardiovascular Medicine, Hiroshima University Graduate School of Biomedical and Health Sciences, Hiroshima University, Hiroshima, Japan; 2 Department of Regeneration and Medicine, Research Center for Radiation Genome Medicine, Research Institute for Radiation Biology and Medicine (RIRBM), Hiroshima University, Hiroshima, Japan; 3 Department of Medicine and Molecular Science, Hiroshima University, Hiroshima, Japan; 4 Department of Regeneration and Medicine, Medical Center for Translational and Clinical Research, Hiroshima University Hospital, Hiroshima, Japan; 5 Department of Integrated Rehabilitation, Hiroshima International University, Hiroshima, Japan; 6 Section of Cardiology, University of Chicago Medical Center, Chicago, IL, United States of America; University of Iowa, United States of America

## Abstract

**Objective:**

Rho-associated kinase (ROCK) signaling pathway has been shown to mediate various cellular functions including cell proliferation, migration, adhesion, apoptosis, and contraction, all of which may be involved in pathogenesis of atherosclerosis. Endogenous nitric oxide (NO) is well known to have an anti-atherosclerotic effect, whereas the exogenous NO-mediated cardiovascular effect still remains controversial. The purpose of this study was to evaluate the effect of exogenous NO on ROCK activity in vascular smooth muscle cells (VSMCs) in vitro and in vivo.

**Methods:**

VSMCs migration was evaluated using a modified Boyden chamber assay. ROCK activities were measured by Western blot analysis in murine and human VSMCs and aorta of mice treated with or without angiotensin II (Ang II) and/or sodium nitroprusside (SNP), an NO donor.

**Results:**

Co-treatment with SNP inhibited the Ang II-induced cell migration and increases in ROCK activity in murine and human VSMCs. Similarly, the increased ROCK activity 2 weeks after Ang II infusion in the mouse aorta was substantially inhibited by subcutaneous injection of SNP.

**Conclusions:**

These findings suggest that administration of exogenous NO can inhibit ROCK activity in VSMCs in vitro and in vivo.

## Introduction

Endogenous nitric oxide (NO) is well known as a pivotal vasodilator component released from the endothelium to regulate vascular tone and maintenance of vascular homeostasis similar to prostacyclin and endothelium-derived hyperpolarizing factor. Endothelial dysfunction, i.e., reduced endothelium-derived NO production, has also been established as an initial step of atherosclerotic process, resulting in future increases of cardiovascular morbidity and mortality [1]. Endogenous NO, produced from L-arginine in the presence of endothelial NO synthase (eNOS) under physiological conditions, e.g., shear stress, stimulates soluble guanylate cyclase and increases production of the second messenger cyclic 3′5′-guanosine monophosphate (cGMP) in vascular smooth muscle cells (VSMCs), which induces the relaxation of vascular tone [2]. In addition, endogenous NO plays diverse roles including inhibition of platelet aggregation, leukocyte adhesion to the vessel wall, and smooth muscle cell proliferation [3]. Endogenous NO, therefore, is an essential signaling molecule for cardiovascular protection by regulating various ranges of cellular and physiological processes.

Rho-associated kinase (ROCK), an immediate downstream target protein of RhoA, has been revealed to be associated with endothelial dysfunction and cardiovascular diseases [4]–[7]. Indeed, several lines of evidence have shown that the RhoA/ROCK signaling pathway mediates various cellular and physiological functions including cell proliferation, migration, adhesion, apoptosis, and contraction [7]–[9], all of which may be involved in the cellular/organ damage and pathogenesis of atherosclerosis. Therefore, ROCK could be a novel therapeutic target for treatment of cardiovascular diseases.

To date, a functional relationship between RhoA/ROCK and NO/cGMP pathways in the vasculature has been reported. Indeed, activation of the RhoA/ROCK pathway has been shown to mediate eNOS mRNA destabilization and eNOS dephosphorylation at Ser1177, leading to the inhibition of eNOS expression and activation, which results in subsequent decrease of NO bioavailability [5], [10]. On the other hand, NO has been shown to phosphorylate RhoA at Ser188, which could prevent its translocation from the cytosol to membrane, resulting in inhibition of RhoA activation [11], [12]. Additionally, Chitaley et al. have demonstrated that endogenous NO-mediated vasodilation could occur through inhibition of the RhoA/ROCK pathway in the rat aorta [13]. However, there is still little information concerning the inhibitory effect of RhoA/ROCK signaling pathway by exogenous, but not endogenous, NO in vivo. In the present study, therefore, we aimed to determine the role of exogenous NO in ROCK activity in VSMCs in vitro and in vivo.

## Methods

### Cell Culture

Isolation and primary culture of VSMCs from wild-type (WT) mice were described previously [14]. At least two independent preparations were used. Human aortic VSMCs were commercially obtained (Cambrex Bio Science Walkersville, Inc., Maryland, USA). To evaluate ROCK activity by Western blotting, cells were treated with a vehicle or an NO donor, sodium nitroprusside (SNP; 40 µmol/L), for 3 hours and stimulated with saline or angiotensin II (Ang II; 40 µmol/L) for the last 1 hour.

### Animal Preparation

WT mice on a C57BL/6J background were purchased from CLEA Japan Inc. (Tokyo, Japan). Animals were anesthetized with isoflurane (50 mg/kg), and a micro-osmotic pump (Alzet model 1002) was subcutaneously implanted. The pumps contained Ang II dissolved in saline, and the infusion rate was 2 mg/kg/day for 14 days. Sham-operated control mice underwent an identical surgical procedure, but with a pump containing saline alone. Both Ang II-infused and control mice were given a subcutaneous injection of SNP dissolved in saline at a dose of 5 mg/kg once a day or saline alone for 14 days, beginning from the day of pump implantation. Ang II-infused mice received drinking water with hydralazine (20 mg/kg/day) to lower blood pressure to the level similar to that in control mice. Before and at 7 days and 14 days after pump implantation, systolic blood pressure and heart rate were noninvasively measured in conscious mice by the tail-cuff method. Systolic blood pressure and heart rate were averaged from two consecutive measurements. Body weight was measured before and 14 days after pump implantation. After measurements, mice were euthanized and perfused with phosphate-buffered saline (PBS) (pH 7.4). The aorta of each mouse was dissected free from the surrounding connective tissue for ROCK activity assay. The experimental procedures and housing conditions for the animals were approved by the Committee of Animal Experimentation, Hiroshima University.

### Assay of cGMP Accumulation

Intracellular cGMP accumulation of isolated murine and human aortic VSMCs were measured 5 minutes after the exposure of saline (control) or SNP (40 µmol/L) using cGMP assay kit according to the manufacturer's instructions (R&D Systems, Inc., Minneapolis, USA) [15]. Data are shown as the ratio to control due to repeated measurements.

### Sample Preparation for ROCK Activity Measurement

ROCK activity was assayed in VSMCs and aortas isolated from mice [16]. VSMCs and mouse aortas were fixed in 10% trichloroacetic acid and 10 mmol/L dichlorodiphenyltrichloroethane. After centrifugation, the cell pellets were stored at −80°C for Western blot analysis.

### Western Blotting

Cells pellets were dissolved in 10 µL of 1 mol/L Tris base and then mixed with 100 µL of extraction buffer (8 mol/L urea, 2% sodium dodecyl sulfate, 5% sucrose and 5% 2-mercaptoethanol). Equal amounts of cell extracts were subjected to 7.5% sodium dodecyl sulfate-polyacrylamide gel electrophoresis and transferred to nitrocellulose membranes. NIH 3T3 cell lysates were used as a positive control and to standardize the results of Western blot analyses from several membranes. Membranes were incubated with rabbit anti-phospho-specific Thr853–myosin-binding subunit (MBS) polyclonal antibody (Biosource Invitrogen, Carlsbad, CA, USA), rabbit anti-MBS polyclonal antibody (Covance Laboratories, Evansville, IN, USA), anti-ROCK2 monoclonal antibody, anti-ROCK1 monoclonal antibody (BD Biosciences, San Jose, CA, USA) or anti-actin monoclonal antibody (Sigma). ROCK activity was expressed as the ratio of phospho-Thr853–MBS in each sample to phospho-Thr853–MBS in each positive control divided by MBS in each sample per MBS in each positive control, as previously described [16].

### Cell Migration Assay

VSMCs migration was evaluated using a modified Boyden chamber assay, as described previously [17], [18]. Briefly, isolated VSMCs were detached mechanically by using a cell scraper, harvested by means of centrifugation, resuspended in 300 µL of the medium for each cell, and counted. Dulbecco's modified Eagle medium (DMEM, Sigma) was used for murine VSMCs, and smooth muscle growth medium-2 (SmGM-2, Lonza) was used for human aortic VSMCs. The 1×10^5^ VSMCs were placed in the upper chamber of a modified Boydenchamber (FluroBlock, Becton Dickinson Biosciences). The chamber was placed in a 24-well culture dish containing each medium for control and each medium with or without Ang II (40 µmol/L) and/or SNP (40 µmol/L). After 24 hours of incubation at 37°C, the lower side of the filter was washed with PBS and fixed with 2% paraformaldehyde. For quantification of cells that had migrated, cell nuclei were stained with 4′,6-diamino-phenylidole (DAPI, Sigma). Migrated cells in the lower chamber were counted manually in the 3 random high-power fields. Each experiment was performed in triplicate.

### Statistical Analysis

Results are presented as mean±SD. P values less than 0.05 were considered to indicate statistical significance. Multigroup comparisons of variables were carried out by one-way ANOVA followed by Bonferroni correction. The data were processed using the software package Stat View V (SAS Institute Inc., Cary, North Carolina, USA).

## Results

### Effects of SNP on cGMP accumulation and cell migration in murine and human aortic VSMCs

**Figure 1:**
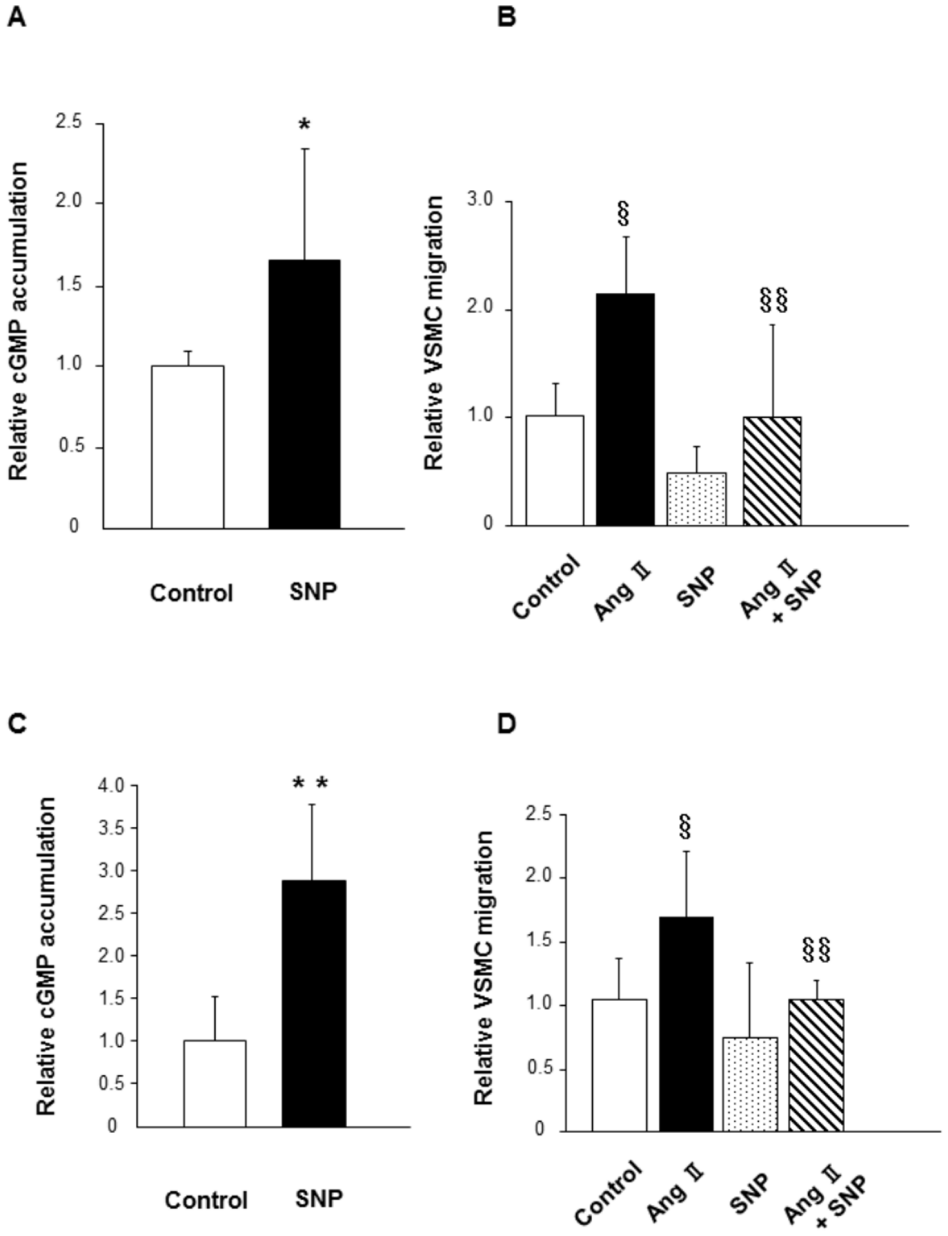
Effect of SNP on cGMP accumulation and cell migration in murine and human aortic VSMCs. **(A)** cGMP accumulation was evaluated after treatment with a vehicle or SNP (40 µmol/L) for 5 minutes in murine VSMCs. n = 6 to 8 in each group. **(B)** Murine VSMCs migration was evaluated using a modified Boyden chamber assay after 24 hours of incubation with a vehicle or SNP (40 µmol/L) and/or Ang II (40 µmol/L). n = 12 to 13 in each group. **(C)** cGMP accumulation was evaluated after treatment with a vehicle or SNP (40 µmol/L) for 5 minutes in human aortic VSMCs. n = 5 to 7 in each group. **(D)** Human aortic VSMCs migration was evaluated using a modified Boyden chamber assay after 24 hours of incubation with a vehicle or SNP (40 µmol/L) and/or Ang II (40 µmol/L). n = 12 in each group. *P<0.05 compared to control; ^§^P<0.0001 compared to control; ^§§^P<0.0001 compared to Ang II. **P<0.01 compared to control.

Initially, we confirmed that administration of SNP, an NO donor, increased the accumulation of cGMP in murine VSMCs (P<0.05 vs. control; Figure 1A). To evaluate the effect of exogenous NO on cell migration, we treated murine VSMCs with or without Ang II and/or SNP. Treatment with Ang II substantially increased cell migration (P<0.0001 vs. control), while co-treatment with SNP inhibited cell migration (P<0.0001 vs. Ang II alone; Figure 1B).

In human aortic VSMCs, we also confirmed that administration of SNP increased the accumulation of cGMP (P<0.01 vs. control; Figure 1C). Similar to the effect of SNP in murine VSMCs, treatment with Ang II substantially increased cell migration (P<0.0001 vs. control), whereas co-treatment with SNP inhibited cell migration (P<0.0001 vs. Ang II alone; Figure 1D).

### Effect of SNP on ROCK activity in murine VSMCs

**Figure 2:**
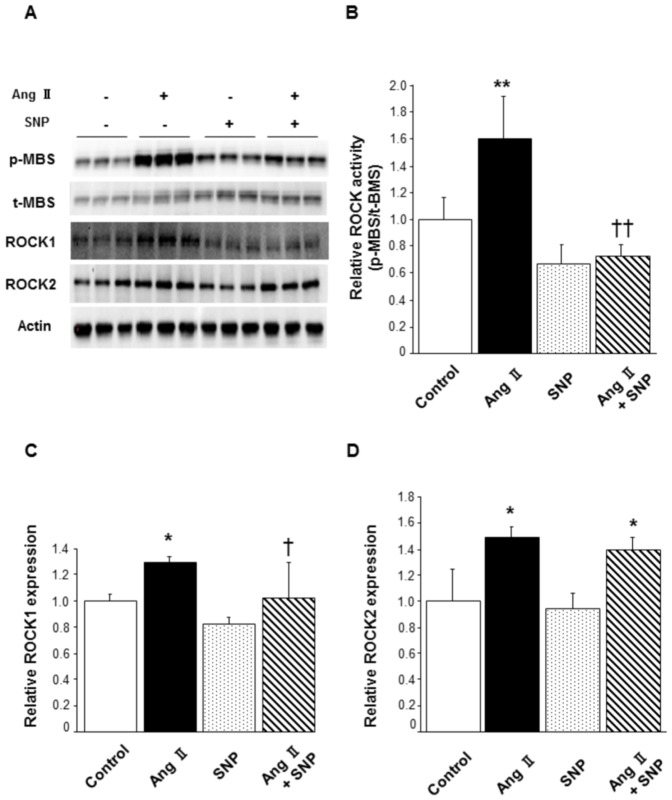
Effect of SNP on Ang II-induced ROCK activation in murine VSMCs. Murine VSMCs were treated with a vehicle or SNP (40 µmol/L) for 3 hours and stimulated with saline or Ang II (40 µmol/L) for the last 1 hour. **(A)** Representative results of Western blot analysis for p-MBS, t-MBS, ROCK1, ROCK2, and actin. **(B)** Quantitative analysis of relative ROCK activity (p-MBS/t-MBS). **(C)** Quantitative analysis of relative ROCK1 expression. **(D)** Quantitative analysis of relative ROCK2 expression. n = 3 in each group. **P<0.01 compared to control; ^††^P<0.01 compared to Ang II; *P<0.05 compared to control; ^†^P<0.05 compared to Ang II.

To evaluate the effect of exogenous NO on ROCK activity, we treated murine VSMCs with or without Ang II and/or SNP. Treatment with Ang II substantially increased ROCK activity (1.60±0.31 in Ang II alone vs. 1.00±0.16 in control, P<0.01; Figure 2B), which was, however, inhibited by co-treatment with SNP (0.73±0.10 in Ang II with SNP, P<0.01 vs. Ang II alone). Treatment with SNP alone did not alter ROCK activity compared to that in control (0.66±0.15 in SNP alone). ROCK1 expression in cells treated with Ang II alone was significantly increased compared to that in control (1.30±0.04 in Ang II alone vs. 1.00±0.05 in control, P<0.05; Figure 2C), and co-treatment with SNP inhibited the increase of ROCK1 expression evoked by Ang II (1.02±0.28 in Ang II with SNP, P<0.05 vs. Ang II alone). ROCK1 expression was not altered by SNP treatment (0.82±0.05 in SNP alone). Concerning ROCK2, treatment with Ang II substantially increased ROCK2 expression compared to that in control with or without SNP co-treatment (1.49±0.09 in Ang II alone, 1.39±0.10 in Ang II with SNP, P<0.05 vs. 1.00±0.25 in control, respectively; Figure 2D). ROCK2 expression was not altered by treatment with SNP alone (0.95±0.11 in SNP alone).

### Effect of SNP on ROCK activity in human aortic VSMCs

**Figure 3:**
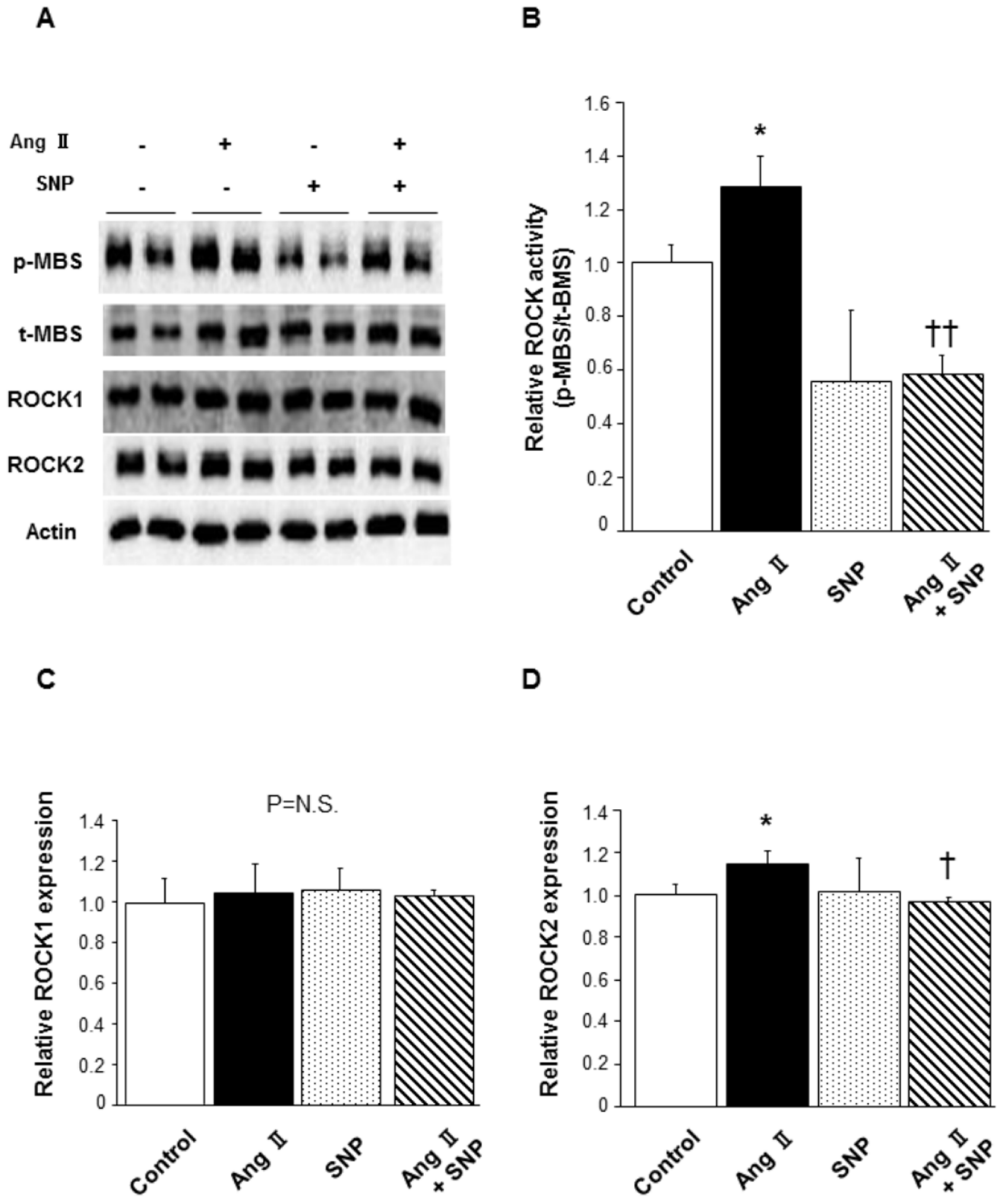
Effect of SNP on Ang II-induced ROCK activation in human aortic VSMCs. Human aortic VSMCs were treated with a vehicle or SNP (40 µmol/L) for 3 hours and stimulated with saline or Ang II (40 µmol/L) for the last 1 hour. **(A)** Representative results of Western blot analysis for p-MBS, t-MBS, ROCK1, ROCK2, and actin. **(B)** Quantitative analysis of relative ROCK activity (p-MBS/t-MBS). **(C)** Quantitative analysis of relative ROCK1 expression. **(D)** Quantitative analysis of relative ROCK2 expression. n = 4 in each group. *P<0.05 compared to control; ^††^P<0.01 compared to Ang II. ^†^P<0.05 compared to Ang II.

In human aortic VSMCs, treatment with Ang II significantly increased ROCK activity (1.28±0.12 in Ang II alone vs. 1.00±0.06 in control, P<0.05; Figure 3B) and the increase in ROCK activity was inhibited by co-treatment with SNP (0.59±0.07 in Ang II with SNP, P<0.01 vs. Ang II alone). ROCK activity was not altered by treatment with SNP alone compared to that in control (0.56±0.27 in SNP alone). There was no alteration of ROCK1 expression in any group (Figure 3C). ROCK2 expression was increased by Ang II treatment (1.15±0.06 in Ang II alone vs. 1.00±0.05 in control, P<0.05), while the increased ROCK2 expression was abolished by co-treatment with SNP (0.97±0.02 in Ang II with SNP, P<0.05 vs. Ang II alone; Figure 3D). Treatment with SNP alone did not alter ROCK2 expression (1.02±0.16 in SNP alone).

### Effect of SNP, an NO donor, on ROCK activity in mice

**Figure 4:**
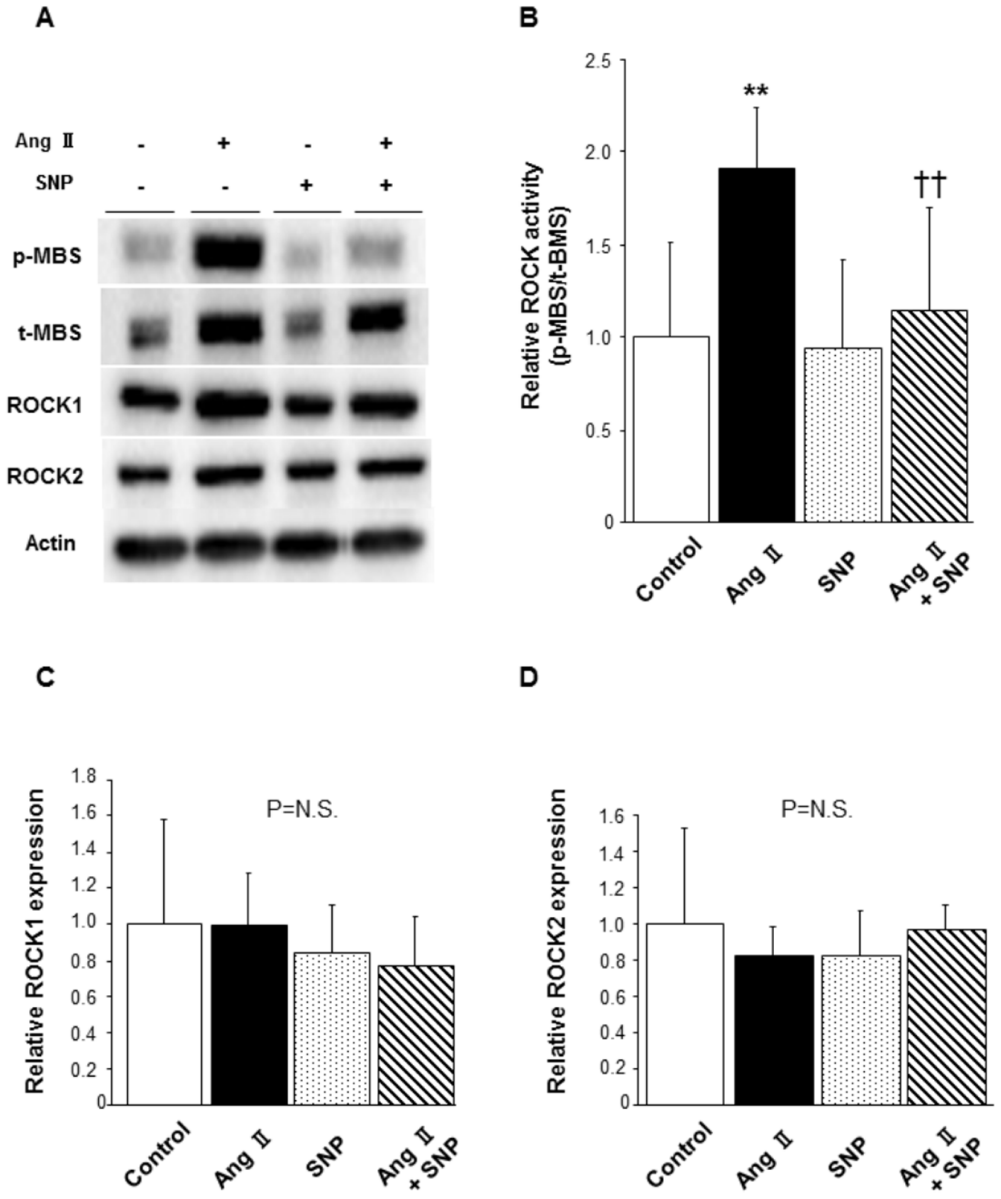
Effect of SNP on Ang II-induced ROCK activation in mouse aortas. Mice were treated with saline or Ang II via implanted micro-osmotic pump infusion (2 mg/kg/day) and treated with saline or SNP via subcutaneous injection (5 mg/kg once a day) for 14 days. Increased blood pressures in Ang II-treated mice were normalized to levels similar to those in mice without Ang II treatment by hydralazine included in drinking water (20 mg/kg/day). **(A)** Representative results of Western blot analysis for p-MBS, t-MBS, ROCK1, ROCK2, and actin. **(B)** Quantitative analysis of relative ROCK activity (p-MBS/t-MBS). **(C)** Quantitative analysis of relative ROCK1 expression. **(D)** Quantitative analysis of relative ROCK2 expression. n = 5–11 in each group; **P<0.01 compared to control; ^††^P<0.01 compared to Ang II.

There was no substantial difference in systolic blood pressure at 7 days and 14 days after pump implantation among the 4 groups, because oral administration of hydralazine lowered the increased blood pressure in Ang II-infused mice to a level similar to that in control mice (Table 1). Also, heart rates were not significantly different among the groups. Body weights in Ang II-infused mice were decreased at 14 days after pump implantation. Western blot analysis demonstrated that ROCK activity was significantly increased in the aortas of Ang II-infused mice compared to that in aortas of control mice (1.92±0.32 in Ang II alone vs. 1.00±0.52 in control, P<0.01; Figure 4B). Ang II-induced increase in ROCK activity was markedly inhibited to the similar level by subcutaneous injection of SNP (1.15±0.56 in Ang II with SNP, P<0.01 vs. Ang II alone). Treatment with SNP alone had no effect on ROCK activity (0.94±0.48 in SNP alone). There were no significant differences in levels of ROCK1 and ROCK2 expression among the groups (Figure 4, C and D).

**Table 1:** Body Weight, Systemic Hemodynamics and Heart Weight/Tibial Length in Control, Ang II-infused, SNP-infused, and A combined Ang II and SNP-infused Mice.

Parameters	Control (n = 5)	Ang II (n = 5)	SNP (n = 8)	Ang II+SNP (n = 11)
Body weight, g				
Before	27.7±1.6	26.7±1.5	27.7±2.7	26.7±1.8
At 14 days	28.3±1.6*	24.4±1.5†	27.2±2.2	24.0±2.1* †
Systolic blood pressure, mmHg				
Before	104±7	102±6	108±11	105±7
At 7 days	106±5	94±20	108±8	110±31
At 14 days	114±9	107±24	103±13	94±29
Heart rate, bpm				
Before	676±40	687±60	680±35	640±72
At 7 days	663±58	699±55	619±80	662±77
At 14 days	645±77	701±56	627±87	661±75
Heart weight/tibial length, mg/mm	6.6±0.2	6.6±0.4	6.1±1.0	6.4±0.4

Ang II indicates angiotensin II; SNP, sodium nitroprusside.

*P<0.05 vs. before,

† P<0.05 vs. control.

All results are presented as mean±SD.

## Discussion

In the present study, we demonstrated that SNP, an NO donor, inhibited Ang II-induced cell migration and ROCK activities in both murine and human VSMCs in vitro. We also revealed in vivo that subcutaneous injection of SNP resulted in a substantial decrease in ROCK activity in the Ang II-infused mouse aorta. These findings demonstrate that administration of exogenous NO can inhibit the RhoA/ROCK signaling pathway in VSMCs in vitro and in vivo.

Accumulating evidence has indicated that the RhoA/ROCK pathway physiologically plays a key role in atherosclerotic lesion formation, vascular inflammation [19], vasoconstriction [20], [21], and hypertrophy [22]. ROCK, therefore, is becoming a key therapeutic target for the prevention of cardiovascular damage. In order to activate ROCK in vitro and in vivo, we used Ang II as a stimulating agent in this study. Previously, it has been shown that Ang II activates the RhoA/ROCK signaling [22], [23] via Ang II type I (AT1) receptor, one of the transmenbrane G protein-coupled receptors. After binding of Ang II, the AT1 receptor interacts with G proteins G_12_-G_13_, and the activated α subunit of G_12_-G_13_ also binds to the regulator of G protein signaling domain in a Rho guanine nucleotide exchange factor, resulting in RhoA and subsequent ROCK activation [24]. In the present study, Ang II significantly increased ROCK activities in murine and human VSMCs, concomitant with Ang II-mediated increase of cell migration which indicates the development of cellular damage and atherosclerotic process. Similarly, murine aortic ROCK activity was also increased by Ang II infusion in vivo. Indeed, there was no substantial difference in blood pressure among the four groups throughout the 14-day study period because hydralazine was orally administered to the mice with Ang II infusion. Furthermore, a previous study has shown that systolic blood pressure decreased only 15–35% in 2–10 min and recovered within 30 min following 2 mg/kg SNP subcutaneous injection in mice [25]. Collectively, Ang II-induced increase in ROCK activity was independent of blood pressure in the present study. We found small but significant Ang II-induced increases in ROCK1 and ROCK2 protein levels in murine VSMCs and ROCK2 protein levels in human aortic VSMCs. Although genetic ablation of ROCK1 or ROCK2 has been reported to be associated with various pathological conditions [16], [26], the pathophysiological significance of the Ang II-induced increases in ROCK protein levels is uncertain. Not an increase in ROCK protein level but an increase in ROCK activity plays a key role in Ang II-induced RhoA/ROCK signaling activation.

The main finding of this study is that exogenous NO inhibits ROCK activities in murine and human VSMCs and mouse aortas. Endogenous NO, released from the endothelium in response to numerous stimuli, is well known to have protective effects against diverse cardiovascular injuries. Indeed, we have found that administration of SNP induced cGMP accumulation and substantially inhibited Ang II-evoked cell migration in vitro, suggesting that exogenous NO could prevent the development of atherosclerotic processes. However, continuous application of exogenous NO is known to evoke the tolerance and subsequent loss of the efficiency, which could potentially mediate extra harmful effects such as increased oxidative stress, endothelial dysfunction, and sympathetic activation [27]. The precise mechanisms underlying nitrate tolerance, however, are not completely understood. The most popular and clinical technique to prevent the tolerance phenomenon is to have a nitrate-free interval. Although intermittent nitrate therapy, which allows a daily nitrate washout interval, cannot provide continuous and uninterrupted therapeutic effects, this regimen has been reported to be effective for preventing nitrate tolerance [28],[29]. In the present study, to minimize the harmful effects evoked by nitrate tolerance in vivo, SNP was subcutaneously injected in mice once a day for intermittent administration. Consequently, we found that ROCK activities in mouse aorta were reduced by exogenous NO administration. In this study, all of the examinations *in vitro* were performed within 24 hours, whereas the examinations *in vivo* were performed at 14 days, viz. we performed acute phase experiments *in vitro* and chronic phase experiments *in vivo*. Nevertheless, both studies demonstrated an inhibitory effect of exogenous NO on ROCK activity, suggesting that administration of exogenous NO may inhibit ROCK activity in VSMCs both in acute and chronic phases.

VSMC is the principal blood vessel component and plays a central role in vessel tone regulation and pathogenesis of atherosclerosis. Indeed, smooth muscle tone is regulated by both intracellular Ca^2+^ concentration and Ca^2+^ sensitivity. Increase in Ca^2+^ concentration initiates activation of Ca^2+^/calmodulin-dependent myosin light chain kinase (MLCK), which subsequently phosphorylates myosin light chain (MLC) and activates myosin ATPase, leading to cellular contraction. On the other hand, Ca^2+^ sensitivity is regulated mainly through ROCK activity [30]. Increased ROCK activity phosphorylates MBS on MLC phosphatase (MLCPh), which leads to MLCPh inactivation and subsequent relative MLCK activation, resulting in MLC phosphorylation and VSMC contraction. In VSMCs, not only endogenous but also exogenous NO could increase cGMP levels, leading to the activation of cGMP-dependent protein kinase type I (cGK I). cGK I lowers Ca^2+^ concentration via phosphorylation of inositol 1,4,5 trisphosphate receptor-associated cGK I substrate (IRAG) and regulator of G-protein signaling 2 (RGS2) [31], [32]. Although ROCK is known to regulate endogenous NO bioavailability via modulation of eNOS expression and phosphorylation [4], [5], little is known about the role of exogenous NO in ROCK activity, especially in vivo. In the present study, we revealed that Ang II-induced increase in ROCK activity in the mouse aortas was abolished by subcutaneous injection of SNP *in vivo*, indicating that exogenous NO inhibits ROCK activity in mouse aortas. Some possible mechanisms by which exogenous NO inhibits ROCK activity in VSMC are postulated. Largiader et al. demonstrated in an *in vitro* study that exogenous NO-mediated cGK I inhibits PDGF-BB-induced human VSMC migration via blockade of the RhoA signaling pathway [33]. It has also been shown that cGK I-induced phosphorylation of RhoA at Ser188 removes activated RhoA from the membrane to the cytosol and increases its interaction with guanine nucleotide dissociation inhibitor [11], [12], which leads to substantial ROCK inhibition, MLCPh activation, and subsequent MLC dephosphorylation. Suzuki et al. reported that the eNOS/NO cascade inhibits ROCK activity through phosphorylation of G_12_-G_13_, the key G proteins crucial for Rho/ROCK signaling activation that interacts with the AT1 receptor [23]. The same mechanisms may be involved in the inhibition of ROCK activity by exogenous NO in mouse aortas. Considering the treatment in patients with cardiovascular diseases in which the RhoA/ROCK signaling is particularly involved, such as spastic angina [20] and stable angina pectoris [34], the clinical view that intermittent administration of organic nitrates cannot only replace the compromised endothelial NO production without nitrate tolerance but also inhibits the RhoA/ROCK pathway is fairly plausible and attractive. Further studies are warranted to investigate whether treatment of patients with cardiovascular diseases with organic nitrate reduces ROCK activities.

In conclusion, we demonstrated the inhibitory effect of exogenous NO on ROCK activity in VSMCs in vitro and in vivo. Administration of exogenous NO for the treatment of cardiovascular diseases with increased ROCK activities, such as stable angina pectoris or vasospastic angina, may have beneficial effects in the prevention of atherosclerosis through inhibition of RhoA/ROCK signaling. Further research into the prognostic importance of the treatment of cardiovascular disease with exogenous NO is needed.
